# Acceptance and safety of femoral versus radial access for percutaneous coronary intervention (PCI): results from a large monitor-controlled German registry (QuIK)

**DOI:** 10.1186/s12872-021-02283-0

**Published:** 2022-01-12

**Authors:** Jörg Reifart, Stefan Göhring, Alexander Albrecht, Winfried Haerer, Benny Levenson, Gerd Ringwald, Patrick Gärtner, Nicolaus Reifart

**Affiliations:** 1 Department of Cardiology, Kerckhoff Heart Center, Benekestr. 2-8, 61231 Bad Nauheim, Germany; 2 DZHK (German Center for Cardiovascular Research), Partner Site RheinMain, Frankfurt am Main, Germany; 3 Geschäftsstelle Qualitätssicherung Invasive Kardiologie, Idstein, Germany; 4 Kardiologische Gemeinschaftspraxis, Berlin, Germany; 5 Herzklinik Ulm, Dr. Haerer und Partner, Überörtliche BAG, Ulm, Germany; 6 Kardiologie im Friedrichspalais, Bruchsal, Germany; 7 Department of Cardiology, Petrus-Krankenhaus, Wuppertal, Germany

**Keywords:** Percutaneous coronary intervention, Radial access, Coronary artery disease, Acute myocardial infarction, BNK, Access site complications

## Abstract

**Background:**

In 2015 and 2018, European Society of Cardiology guidelines for percutaneous coronary intervention (PCI) favoring radial access over femoral access were published. These recommendations were based on randomized trials suggesting that patients treated radially experienced reduced bleeding complications and all-cause mortality. We aimed to assess acceptance and results of radial access in a real-world scenario by analyzing all PCI cases in the Quality Assurance in Invasive Cardiology (QuIK) registry.

**Methods:**

The QuIK registry prospectively collects data on all diagnostic and interventional coronary procedures from 148 private practice cardiology centers in Germany. Major adverse cardiac and cerebrovascular events (MACE) were defined as myocardial infarction, stroke, or death during hospitalization.

**Results:**

From 2012 to 2018, 189,917 patients underwent PCI via either access method. The rate of radial approach steadily increased from 13 to 49%. The groups did not differ significantly with respect to age or extent of coronary disease. Femoral approach was significantly more common in patients with ST elevation myocardial infarction and cardiogenic shock. Overall, there were significant differences in MACE (radial 0.12%; femoral 0.24%; *p* < 0.0009) and access site complications (radial 0.2%; femoral 0.8% (*p* < 0.0009).

**Conclusion:**

Our data reveals an increase in use of radial access in recent years in Germany. The radial approach emerged as favorable regarding MACE in non-myocardial infarction patients, as well as favorable regarding access site complication regardless of indication for percutaneous intervention.

## Introduction

In the last 10 years, several randomized trials have made the case for radial access over femoral access in coronary angiography as well as PCI [1–3]. While the majority of data from randomized controlled trials shows lower complication rates with radial access, there are some data that suggest no difference [1, 4]. Consequently, the guidelines of the European Society of Cardiology (ESC) strongly recommended the radial approach as the default access for both diagnostic and interventional procedures [5–7].

In Germany, roughly 881,000 diagnostic coronary angiographies and 378,000 PCI are performed every year, but there is no information available on the rate of transradial and transfemoral approach [8, 9].

We analyzed data from a database of German private practices: the QuIK registry (Quality Assurance in Invasive Cardiology).

In Germany, about 9% of coronary angiographies and 4% of all PCI are performed by highly experienced cardiologists in private practice in cooperation with an affiliated hospital.

Our goal was to elucidate the current situation in Germany with respect to guideline adherence and impact on patient outcomes in a subgroup of interventional centers.

We compiled and assessed data from all diagnostic angiograms, interventions in acute coronary syndrome (ACS) (subdivided by ST elevation myocardial infarction (STEMI), non-ST elevation myocardial infarction (NSTEMI) and unstable angina), as well as stable coronary artery disease (SCAD).

## Method

### QuIK registry

All data were gathered from the QuIK registry, a monitor-controlled observational online registry that has collected data of all invasive and interventional coronary procedures from 149 centers of cardiologists in private practice in Germany since 1996 [10].

Immediately upon coronary angiography or PCI, data are entered into an electronic file that anonymously reports to the QuIK registry.

Data for diagnostic angiograms, acute procedures (UA, STEMI, NSTEMI), and non-acute procedures (SCAD) from 2012 until 2018 were analyzed for this retrospective study.

### Monitoring

Each center and interventionalist is monitored yearly on site by a random selection and unblinding of 5–20 cases to double-check data entry. If a single center has multiple interventionalists, each is checked separately. Registry inconsistencies found during monitoring lead to a warning. A center is excluded from the registry if it has received three warnings.

### Invasive approach

Access site choice was left to the discretion of each operator.

Antiplatelet regime and heparinization as well as access site treatment after cannulation were performed following institutional protocols.

If coronary angiogram revealed an indication for ad-hoc PCI, values for the diagnostic angiogram and PCI were entered as separate procedures, with complications being assigned to the PCI. All data, including complications, were entered both immediately after the intervention and at the patient’s discharge of hospital, if applicable. Major bleeding was defined as hemorrhage with drop in hemoglobin of > 3 g/L measured the day after the procedure. Severe access site complications were assumed if they resulted in in prolonged hospital stay or required intervention or surgery (TIMI class 2–4).

MACE was defined as myocardial infarction (new Q-waves or CK rise to > 3 × upper limit with CKMB levels > 10% of CK), stroke or death.

Procedural success was defined as < 30% residual diameter stenosis of all treated lesions at the end of the procedure as assessed by visual angiographic inspection or QCA (quantitative coronary angiography) and absence of any MACE [11].

Cardiogenic shock was defined as systolic blood pressure < 90 mmHg or need for catecholamine therapy in the setting of myocardial infarction.

Complications were documented and registered until discharge from the hospital. Data of outpatient catheterizations were only recorded for the same day.

### Statistics

Values are presented as mean ± standard deviation.

Group differences were compared using the Student’s *t* test, z-test and Mann–Whitney-Wilcoxon Test. *P* values of < 0.05 were considered significant. R version 3.6.1, with the corresponding stats package was used for statistical calculations.

## Results

### Study population

A total of 189,917 interventional procedures with either radial or femoral access, performed by 448 interventionalists were entered into the registry from 2012 until 2018.

56,198 were procedures in patients with acute coronary syndrome and 133,719 procedures were considered elective interventions (Table 1).

**Table 1:** Case characteristics

	Radial, n = 46,687 (24.6%)	Femoral, n = 143,230 (75.4%)	*p*
Age	67.70 ± 10.56	67.68 ± 10.97	0.14 ns
Cardiogenic shock	0.4% (n = 202)	1.7% (n = 2379)	< 0.0009***
Prior CABG	2.3% (n = 1074)	2.1% (n = 3024)	0.015*
SCAD	78.1%	67.9%	< 0.0009***
STEMI	2.8%	8.0%	< 0.0009***
NSTEMI	6.6%	11.9%	< 0.0009***

Z test/Mann–Whitney–Wilcoxon Test

Extremely significant ****; Extremely significant ***; Very significant **; Significant *; Not significant ns

Patients not treated via the femoral or radial approach (e.g. brachial artery access) were excluded from the analysis.

In almost 1000 monitoring reports with several thousand double-checked cases, there have been only minor inconsistencies like questionable pre-procedure stress-test documentation or divergent stenosis grading. Since 1998, rejection of a certificate happened in two cases.

### Patient characteristics

While age and extent of coronary disease was similar in both groups, patients with myocardial infarction were more commonly treated femorally, while surprisingly patients with history of coronary artery bypass grafting (CABG) were more commonly treated radially (Table 1).

### Frequency of radial and femoral access

**Fig. 1:**
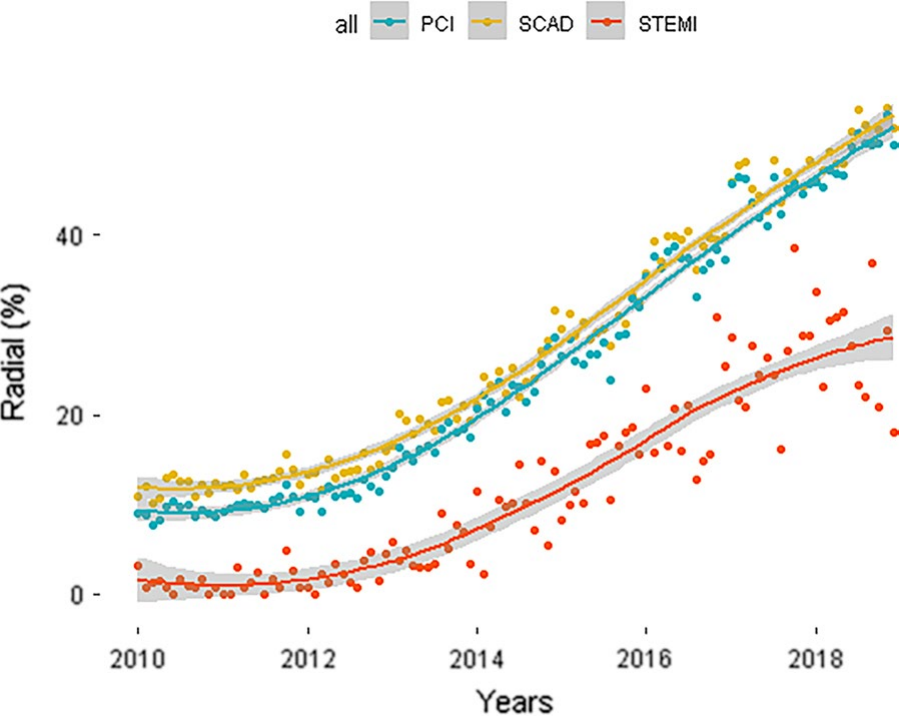
Increase of radial access over time

Application of radial access has increased annually from 13% in 2012 to 48.8% for PCI and 51.5% for diagnostic angiograms over the course of the 6 year study period (Fig. 1). Radial access was more frequently adopted for elective procedures (SCAD), and least frequent in acute PCI of STEMI (Table 1).

PCI was performed in 2581 patients with cardiogenic shock, 7.2% via radial, and 92.2% via femoral route.

We found that the proportion of centers which only used femoral access (0% radial access) decreased from 68% in 2010 to 32% by 2018.

### Procedural data

Procedural success rate was achieved in 92.6% of radial and 91.6% of femoral cases (*p* < 0.001) (Table 2).

**Table 2:** PCI success rates

	Radial (%)	Femoral (%)	*p*
All PCI	92.6	91.6	< 0.0009***
SCAD	92.3	91.7	< 0.0009***
NSTEMI	95.3	92.3	< 0.0009***
STEMI	92.2	91.2	0.073
Unstable angina	94.3	91.2	< 0.0009***
Cardiogenic shock	84.1	86.5	0.4

Pearson’s Chi-squared test with Yates’ continuity correction

Overall, the complication rate was low (MACE radial/femoral 0.12%/0.24%, *p* < 0.001) and there was a trend of reduced complications over time (Table 3).

**Table 3:** Procedural results and complications

All PCI	Radial 24.6% (n = 46,687)	Femoral 75.4% (n = 143,230)	
Procedure time (min)	35.51 ± 24.06	37.99 ± 25.42	< 0.0009***
Fluoroscopy time	8.26 ± 7.96	8.30 ± 8.99	0.45
Contrast dye (ml)	68.32 ± 41.30	77.01 ± 51.66	< 0.0009***
Dose area product (Gycm^2^)	27.75 ± 33.24	33.42 ± 40.6	< 0.0009***
Major bleeding	0.07 (n = 31)	0.2% (n = 280)	< 0.0009***
MACE overall	0.12% (n = 54)	0.24% (n = 342)	< 0.0009***
Myocardial infarction	0.04% (n = 18)	0.03% (n = 49)	0.77
TIA/stroke	0.03 (n = 13)	0.03% (n = 49)	0.95
Death	0.05% (n = 23)	0.18% (n = 256)	< 0.0009***
Access site complication overall	0.2% (n = 108)	0.8% (n = 1036)	< 0.0009***
All SCAD	36,446 (27.3%)	97,273 (72.7%)	
Procedure time (min)	70.2 ± 42.4	79.9 ± 54.4	< 0.0009***
Fluoroscopy time	8.21 ± 7.99	8.35 ± 9.19	< 0.009**
Contrast dye (ml)	70.3 ± 42.3	80 ± 54.3	< 0.0009***
Major bleeding	0.07% (n = 24)	0.15% (n = 149)	< 0.0009***
MACE	0.1% (n = 37)	0.09% (n = 89)	0.68
Myocardial infarction	0.04% (n = 16)	0.04% (n = 37)	0.58
TIA/stroke	0.03% (n = 12)	0.02% (n = 21)	0.33
Death	0.3% (n = 9)	0.03% (n = 32)	0.56
Access site complication	0.02% (n = 91)	0.07% (n = 726)	< 0.0009***
All NSTEMI	3.067 (15.2%)	17.062 (84.8%)	
Procedure time (min)	38.9 ± 25.4	40.3 ± 26.4	< 0.009**
Fluoroscopy time	9.47 ± 8.5	8.61 ± 8.72	< 0.0009***
Contrast dye (ml)	66.2 ± 39.2	73.8 ± 45.1	< 0.0009***
Major bleeding	0.16 (n = 5)	0.32% (n = 54)	0.20
MACE in NSTEMI	0.16% (n = 5)	0.36% (n = 62)	0.11
Myocardial infarction	0.02% (n = 1)	0.03% (n = 4)	1
TIA/stroke	0.0% (n = 0)	0.04% (n = 7)	0.55
Death	0.13% (n = 4)	0.30% (n = 52)	0.15
Access site complication NSTEMI	0.2% (n = 7)	0.8% (n = 131)	< 0.009**
All STEMI	1.578 (11%)	12.821 (89%)	
Procedure time (min)	57.9 ± 34.6	61.6 ± 38.9	< 0.0009***
Fluoroscopy time (min)	8.69 ± 8.54	8.12 ± 8.56	0.02*
Contrast dye (ml)	57.9 ± 34.6	61.6 ± 38.9	< 0.009**
Major bleeding	0.06 (n = 1)	0.39% (n = 50)	0.066
MACE in STEMI	0.6% (n = 10)	1.1% (n = 141/12821)	0.11
Myocardial infarction	0.0% (n = 0)	0.05% (n = 6)	0.84
TIA/stroke	0.0% (n = 0)	0.01% (n = 6)	0.84
Death	0.06% (n = 10)	1.0% (n = 129)	0.196
Access site complication STEMI	0.06% (n = 1)	0.7% (n = 94)	< 0.009**
All unstable angina	5.596 (25.8%)	16.074 (74.2%)	
Procedure time (min)	31.8 ± 22.3	37.3 ± 25.6	< 0.009**
Fluoroscopy time	7.79 ± 7.21	7.76 ± 8.2	0.8
Contrast dye (ml)	59.9 ± 35.2	73.9 ± 46.6	< 0.009**
Major bleeding	0.02 (n = 1)	0.17% (n = 27)	0.013
MACE in unstable angina	0.03% (n = 2)	0.3% (n = 50)	< 0.0009***
Myocardial infarction	0.02% (n = 1)	0.02% (n = 3)	1
TIA/stroke	0.02% (n = 1)	0.02% (n = 3)	1
Death	0.0% (n = 0)	0.27% (n = 44)	< 0.0009***
Access site complication in unstable angina	0.02% (n = 9)	0.05% (n = 85)	< 0.0009***
Cardiogenic shock	202 (7.8%)	2379 (92.2%)	
Major bleeding	0 (n = 0)	0.084% (n = 2)	1
MACE	5.4% (n = 11)	6.9% (n = 165)	0.5
Myocardial infarction	0.0% (n = 0)	0.04% (n = 1)	1
TIA/stroke	0.0% (n = 0)	0.0% (n = 0)	1
Death	5.4% (n = 11)	6.89% (n = 164)	0.52

On further analysis, we found that 165 (48%) of 342 femorally treated patients with MACE had cardiogenic shock. Without this subgroup there was no significant difference in MACE in all PCIs (MACE radial/femoral 0.09%/0.13%, *p* = 0.08).

## Discussion

The 2018 ESC guidelines on myocardial revascularization state that radial approach should be the preferred method of access in primary PCI if performed by an experienced radial operator [7, 12]. Our large-scale multisite registry shows a steady increase in transradial procedures during recent years in Germany, although femoral access is still predominant at most sites.

Complication and MACE rates were very low in both radial and femoral access groups. Radial access was associated with significantly lower access site complications across all indications for PCI. Interestingly, almost 50% of MACE in the femoral group occurred in patients with cardiogenic shock.

Surprisingly, the femoral approach was not associated with shorter fluoroscopy and procedural times. To the contrary, we found a small difference in favor of the radial approach.

Equally surprising is the higher success rate with the radial approach in all groups except cardiogenic shock and STEMI; whether this is due to differences in lesion complexity or other factors cannot be concluded from the available data.

The low rates of MACE for both radial and femoral approach patients in our study were seen in all PCI scenarios including STEMI. Here, we observed lower rates for MACE in STEMI than those reported in the STEMI RADIAL trial (30-day MACE rates of 0.6% for radial and 1.1% for femoral in our study, compared to 3.5% and 4.2%, respectively) [3]. This difference is likely to do a combination of factors.

Limited follow-up (in-hospital), possible selection bias (referral of complex and multivessel disease to centers with in-house heart surgery) and differences in operator experience. Even with the quality assurance monitoring of the QuIK registry, complete data entry and rigorous follow-up comparable to thoroughly controlled trials cannot be guaranteed.

Previous studies comparing radial and femoral approaches did not follow an established protocol for femoral puncture and sheath removal (vascular closure device/compression gauze). Techniques for establishing vascular access and for access site closure are highly variable among operators and can, if not performed correctly, contribute relevantly to access site complications.

Methods of sheath removal and compression or use of vascular closure device were performed according to each site’s own protocols and were not reported in our registry. Another limitation is the lack of reporting regarding failed access attempts (switching from one site to another).

Regarding radial access complications, postinterventional radial artery closure (a meaningful complication that may occur in up to 10% of cases) was not evaluated in our registry nor in any of the randomized comparisons [13].

## Conclusion

The data from our registry shows a slow but increasing adoption of the radial approach in German private practice. Data on complications and results suggests a clinical advantage of radial over femoral approach.

While the femoral approach in the hand of experienced operators is safe and should not be fully abandoned, since it may be advantageous in complex, high risk, or emergent procedures, radial access should be encouraged as the default access [14–16].

Further studies with strict access site protocols (e.g. ultrasound guided access) and post-interventional screening for vascular complications including radial artery injury are needed to compare these PCI approaches more rigorously.

## Data Availability

Data can be made available upon request.

## Abbreviations

ACS: Acute coronary syndrome
CABG: Coronary artery bypass grafting
ESC: European Society of Cardiology
MACE: Major adverse cardiac and cerebrovascular events
NSTEMI: Non-ST elevation myocardial infarction
PCI: Percutaneous coronary intervention
QCA: Quantitative coronary angiography
QuIK: Quality Assurance in Invasive Cardiology
STEMI: ST elevation myocardial infarction
SCAD: Stable coronary artery disease
UA: Unstable angina
